# Enhanced Lipid Oxidation and Maintenance of Muscle Insulin Sensitivity Despite Glucose Intolerance in a Diet-Induced Obesity Mouse Model

**DOI:** 10.1371/journal.pone.0071747

**Published:** 2013-08-12

**Authors:** Karin E. Trajcevski, Hayley M. O’Neill, David C. Wang, Melissa M. Thomas, Dhuha Al-Sajee, Gregory R. Steinberg, Rolando B. Ceddia, Thomas J. Hawke

**Affiliations:** 1 Department of Pathology and Molecular Medicine, McMaster University, Hamilton, Ontario, Canada; 2 Department of Medicine, McMaster University, Hamilton, Ontario, Canada; 3 Muscle Health Research Centre, York University, Toronto, Ontario, Canada; INSERM/UMR 1048, France

## Abstract

**Background:**

Diet-induced obesity is a rising health concern which can lead to the development of glucose intolerance and muscle insulin resistance and, ultimately, type II diabetes mellitus. This research investigates the associations between glucose intolerance or muscle insulin resistance and tissue specific changes during the progression of diet-induced obesity.

**Methodology:**

C57BL/6J mice were fed a normal or high-fat diet (HFD; 60% kcal fat) for 3 or 8 weeks. Disease progression was monitored by measurements of body/tissue mass changes, glucose and insulin tolerance tests, and *ex vivo* glucose uptake in intact muscles. Lipid metabolism was analyzed using metabolic chambers and *ex vivo* palmitate assays in intact muscles. Skeletal muscle, liver and adipose tissues were analyzed for changes in inflammatory gene expression. Plasma was analyzed for insulin levels and inflammatory proteins. Histological techniques were used on muscle and liver cryosections to assess metabolic and morphological changes.

**Principal Findings/Conclusions:**

A rapid shift in whole body metabolism towards lipids was observed with HFD. Following 3 weeks of HFD, elevated total lipid oxidation and an oxidative fiber type shift had occurred in the skeletal muscle, which we propose was responsible for delaying intramyocellular lipid accumulation and maintaining muscle’s insulin sensitivity. Glucose intolerance was present after three weeks of HFD and was associated with an enlarged adipose tissue depot, adipose tissue inflammation and excess hepatic lipids, but not hepatic inflammation. Furthermore, HFD did not significantly increase systemic or muscle inflammation after 3 or 8 weeks of HFD suggesting that early diet-induced obesity does not cause inflammation throughout the whole body. Overall these findings indicate skeletal muscle did not contribute to the development of HFD-induced impairments in whole-body glucose tolerance following 3 weeks of HFD.

## Introduction

An unhealthy lifestyle including a high fat diet (HFD) has become common in Western societies and contributes to obese, insulin resistant states such as pre-diabetes, which if left untreated, can progress to Type 2 Diabetes Mellitus (T2DM) [1]–[3]. It is estimated that 2.4 million Canadians will have T2DM by 2016 [4] with excessive body mass and inactivity accounting for almost 90% of all new cases of T2DM [5]. Given the importance of skeletal muscle to our overall physical and metabolic capacities [6]–[14], improving our understanding of the changes to muscle health during the development of obese, insulin resistant states will advance the development of therapeutic strategies for those individuals.

While the exact cause of muscle insulin resistance with the development of diet-induced obesity is still unknown, one proposed mechanism is elevated delivery of free fatty acids (FFAs) in conjunction with impaired lipid metabolism within skeletal muscle leading to a build-up of intramyocellular lipids (IMCL) and associated lipid derivatives [15]–[17]. Our lab previously reported that mice fed a HFD (60% kcal from fat) for 8 weeks exhibited obesity and muscle insulin resistance accompanied by impaired muscle lipid oxidation and excessive IMCL deposition [18]. Interestingly, despite significant reductions in glucose and palmitate oxidation rates, histological/immunofluorescent analysis of skeletal muscle from HFD fed mice revealed a significant shift towards an oxidative phenotype. This paradoxical observation led us to hypothesize that the shift towards a more oxidative phenotype was an early, adaptive response to the HFD in order to enhance lipid utilization. However, the chronic nature of the diet led to a pathological situation where substrate utilization could not match substrate delivery [18]. Previous reports of rodents fed a similar diet high in fat are equivocal on whether an early adaptive oxidative shift is present [15], [19]–[22] and whether muscle lipid oxidation is altered [15], [19], [20]. However, differences in rodent models, HFD composition and timing of measurements complicate a direct comparison between these studies.

In addition to alterations in lipid metabolism, inflammation is also linked to insulin resistance development. In particular, inflammation in adipose tissue is being extensively investigated as a mechanism contributing to glucose intolerance and insulin resistance in pre-diabetes and T2DM [15], [23], [24]. However, the extent to which inflammation in alternative tissues like liver and skeletal muscle contributes to the initial development of glucose intolerance and tissue specific insulin resistance is unclear as the inflammatory state is often assessed once obesity and insulin resistance is well underway. Furthermore, it is important to directly assess muscle insulin resistance as it is often implied by the presence of glucose intolerance although the two are not always present at the same time during disease development.

The main focus of the present study was on the impact of three weeks of HFD (60% kcal fat) on skeletal muscle morphology, metabolism and insulin resistance development. The overall purpose of our experiments was to determine if there are early positive whole body or muscle-specific adaptations to lipid handling and if changes in inflammatory state and/or lipid metabolism are associated with the development of glucose intolerance and insulin resistance. Our results demonstrate an early elevation in the processing of lipids that we believe leads to the attenuation of muscle insulin resistance following 3 weeks of HFD. Furthermore our data serves to clarify the associations between inflammation, ectopic lipid deposition and glucose intolerance or muscle insulin resistance.

## Results

### HFD Increases Body Mass and Fat Mass of Mice after 3 and 8 Weeks

HFD increased body mass following 3 (∼1.2 fold) or 8 (∼1.4 fold) weeks compared to mice on a normal diet (ND; Table 1). Epididymal fat pad mass was also elevated with HFD compared to ND after 3 weeks (∼ 3.5 fold) and 8 weeks (∼ 4 fold) even though ND fat pad mass increased from 3 to 8 weeks which highlights the extent of obesity in the HFD group. Lower limb muscle mass was unaltered after 3 and 8 weeks of HFD (Table S1).

**Table 1:** Body Composition.

	Body Mass (g)		Fat Mass (mg)	
	ND	HFD	ND	HFD
**Week 0**	25.27±0.35 (N = 11)	25.37±0.45 (N = 12)	–­­	–
**Week 3**	26.60±0.29 (N = 6)	31.52±0.77 (N = 6)* **^#^**	215.14±23.39 (N = 6)	766.87±83.66 (N = 6)*
**Week 8**	29.60±0.92 (N = 5)**^#^**	40.78±2.37 (N = 6)* **^#^**	309.16±20.11 (N = 5) **^#^**	1222.88±72.73 (N = 6)* **^#^**

Body mass and fat mass for normal diet (ND) and high-fat diet (HFD) mice throughout diet intervention up to 8 weeks. Body mass for mice from both 3 and 8 week time-points was included for the 0 week time-point. Data are means ± SEM. Two-way ANOVAs were performed separately for body mass and fat mass, p<0.05,

* vs. ND, ^#^vs. week 0 for body mass or vs. week 3 for fat mass.

### HFD Results in Rapid Increase in Lipid Metabolism

**Figure 1:**
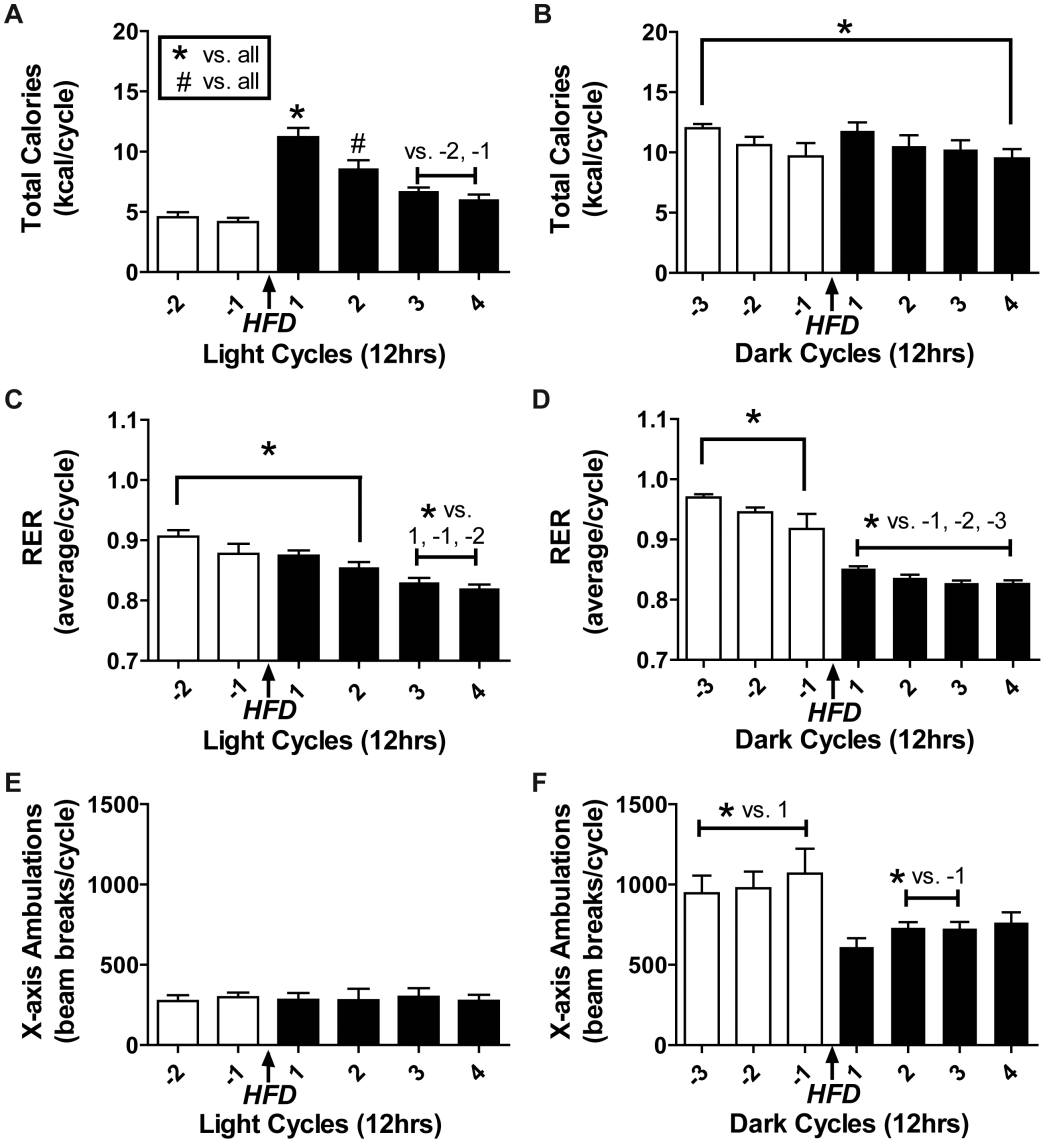
Metabolism related changes during HFD transition. Mice in CLAMS were switched from ND to HFD at the beginning of a light cycle. (A) Light cycle and (B) dark cycle calories consumed per 12 hr cycle (N = 7). (C) Light cycle and (D) dark cycle respiratory exchange ratio (RER; VC0_2_:VO_2_). (E) Light and (F) dark cycle average movement (x-ambulations) per 12 hour cycle. Data are mean ± SEM. Statistics: Repeated measures one-way ANOVA with Tukey’s multiple comparison test, p<0.05. ND (white bars), HFD (black bars). Average CLAMS measurements per cycle are total (A–B) or means (C–F) of measurements taken every 20 minutes with 1 mouse/CLAMS cage, N = 8 unless otherwise noted. Light cycle = 07∶00–19∶00, dark cycle = 19∶00–07∶00.

In an effort to understand the progression of changes in lipid oxidation in response to HFD consumption, we quantified the initial whole body metabolic response using the comprehensive laboratory animal monitoring system (CLAMS). Body mass increased in the CLAMS after 4 days of HFD, but not after an initial 4 days of ND (Figure S1). While total calories during the light cycles (Figure 1A) increased with HFD, total calories during the dark cycles remained relatively constant (Figure 1B). To determine if total calories consumed would differ when corrected for weight gain on HFD, statistics were performed on values from the last day of ND (day -1) and HFD (day 4) relative to body mass at these times. Consistent with initial findings there was still a greater amount of total calories consumed during light cycles following HFD (Day -1∶16.65±1.33 kcal/12 hr cycle/body mass (g); Day 4∶23.05±1.73 kcal/12 hr cycle/body mass (g); t-test, p<0.05) and no difference during dark cycles (Day -1∶38.34±3.81 kcal/12 hr cycle/body mass (g); Day 4∶36.25±3.18 kcal/12 hr cycle/body mass(g); t-test, p<0.05). HFD was introduced after a dark cycle (07∶00) and a drop in the respiratory exchange ratio (RER) occurred during the subsequent dark cycle and the second light cycle (Figure 1C, D). Light cycle activity level (Figure 1E) and exploratory activity (Figure S1) were unaltered with HFD. Dark cycle activity level (Figure 1F) declined temporarily during the first three days of HFD and HFD exploratory activity (Figure S1) fluctuated slightly such that there were differences compared to the initial measurement day (day -3) but not the last two days of ND. In summary, upon commencement of HFD, it took 12–24 hours for metabolism to significantly shift towards greater lipid utilization.

### Increased Number of Oxidative Fibers and Functional Lipid Oxidation in Muscle Following 3 Weeks of HFD

**Figure 2:**
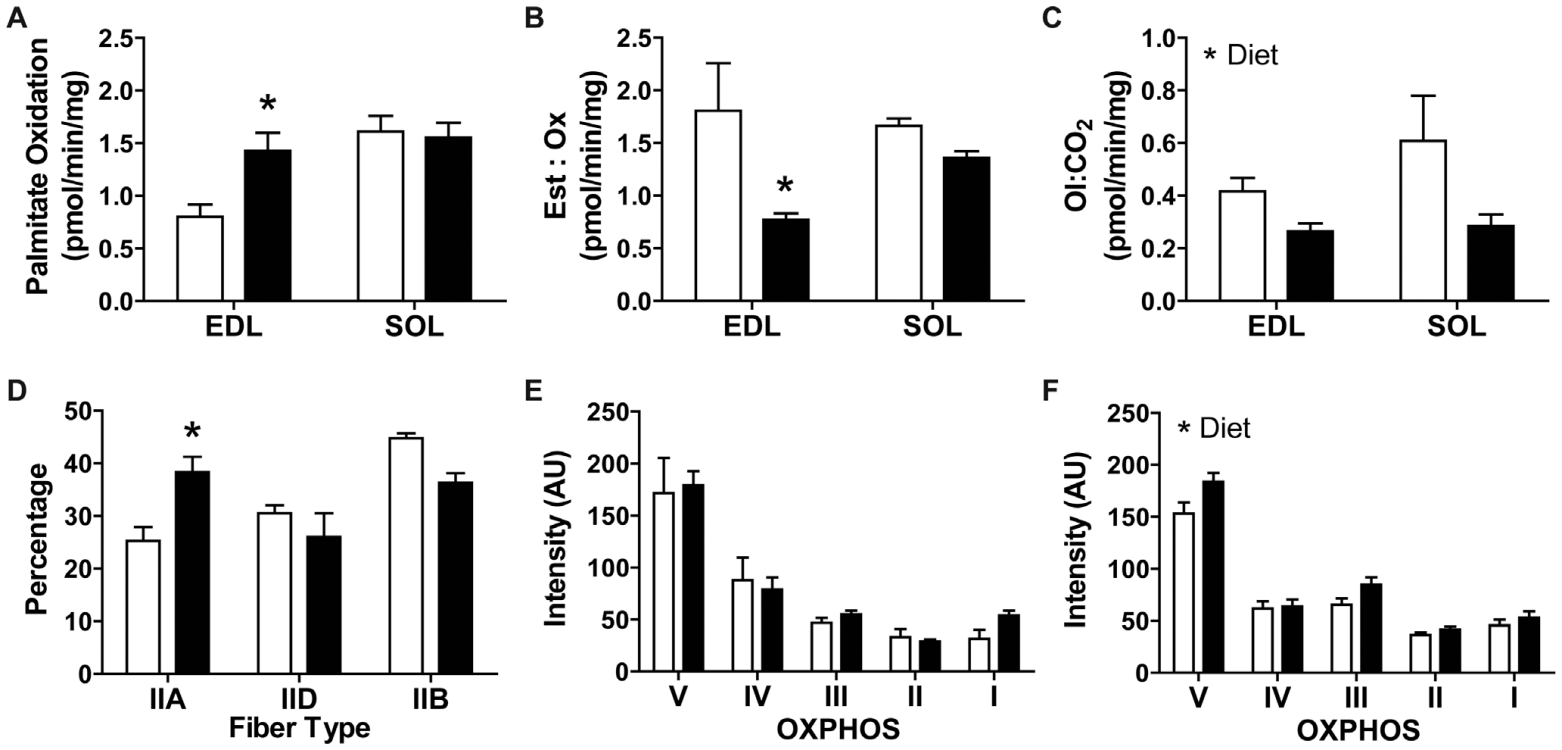
Measures of oxidative metabolism in muscle. Following 3 weeks of diet intervention and using palmitate as a substrate EDL and soleus muscle (A) total oxidation [CO2+ oxidative intermediates (OI)], (B) relative esterification (Est) to TAG and DAG vs. total oxidation (Ox) and (C) incomplete (oxidative intermediates, OI) vs. complete (carbon dioxide, CO_2_) oxidation (N = 4 in duplicate) were assessed. (D) Fiber type percentage in the oxidative area of the GP muscle following 3 weeks of HFD. Type I fibers were not included in the analysis due to the minimal content in GP muscle. Protein analysis of complexes involved in the electron transport chain (ETC; complexes I–V) in whole GP muscle homogenates after (E) 3 weeks and (F) 8 weeks of HFD. Data are mean ± SEM. Statistics: (A–F) Two-way ANOVA with Bonferonni post-hoc test, *p<0.05. ND, white bars; HFD, black bars. Diacylglycerol (DAG), triacylglycerol (TAG).

Our investigation into fatty acid handling in whole muscle demonstrated that the mixed fiber-type EDL muscle (49% fast-glycolytic, 51% fast-oxidative glycolytic), but not the highly oxidative soleus muscle (42% fast-oxidative glycolytic and 58% slow oxidative) [25] exhibited enhanced total palmitate oxidation after 3 weeks of HFD (Figure 2A). The EDL muscle also exhibited less relative esterification than total oxidation with HFD while the soleus did not demonstrate a shift towards utilization of fatty acids, away from storage (Figure 2B). There was no difference in palmitate uptake into either EDL or soleus muscle after 3 weeks of HFD (EDL: ND 2.045±0.098, HFD 2.510±0.349 pmol/min/mg; Soleus: ND 4.307±0.482, HFD 3.739±0.352 pmol/min/mg). Both EDL and soleus muscles exhibited a decrease in the relative amount of incomplete (oxidative intermediates, OI) to complete (CO_2_) β-oxidation of palmitate with HFD (Figure 2C).

Potential mechanisms to explain the increased total palmitate oxidation in mixed fiber-type muscles could include an increase in mitochondrial density or an oxidative fiber-type shift which were investigated in the mixed fiber-type gastrocnemius/plantaris (GP) muscle complex. The density of the oxidative protein succinate dehydrogenase (SDH) in a subset of fast oxidative glycolytic fibers, termed IIA fibers, was not different in the oxidative area of the GP muscle (ND, 1.094±0.067 AU, N = 4; HFD, 1.020±0.148 AU, N = , minimum 15 IIA fibers per muscle). An oxidative fiber-type shift began by 3 weeks of HFD as demonstrated by a greater percentage of IIA fibers within the oxidative area of the muscle (Figure 2D). Since the GP muscle is predominantly fast-glycolytic [26], it is not surprising that our detailed assessment of fiber type oxidative shift was only detectable by protein (Western blot) analysis of complexes involved in the electron transport chain (OXPHOS proteins; complexes I–V) after 8 weeks of HFD (Figure 2E, F).

### Skeletal Muscle is not Insulin Resistant Following 3 Weeks of HFD Despite Glucose Intolerance

**Figure 3:**
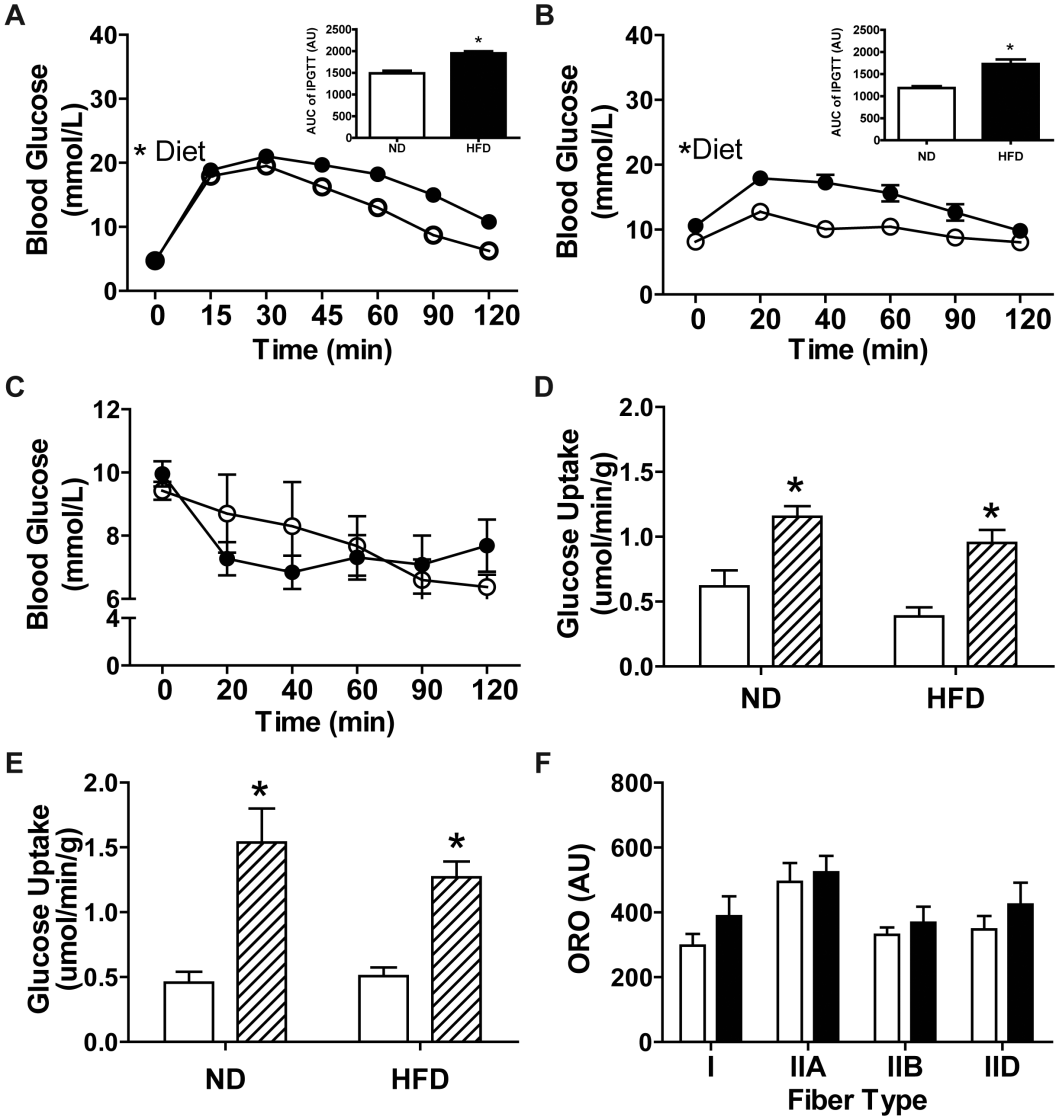
Indices of glucose intolerance and muscle insulin resistance following 3 weeks of HFD. IPGTT performed after a (A) 16 hr fast (N = 6) or (B) 6 hour fast (N = 4 ND, 6 HFD) with inset of area under the curve (AUC) graphs. Legend for A–C: ND, open circles; HFD, closed circles. (C) IPITT performed after a 6 hour fast (N = 4 ND, 6 HFD). Insulin stimulated glucose uptake (basal, white bars; insulin, hatched bars) in isolated (D) EDL (N = 4) and (E) soleus muscles (N = 4 ND, 6 HFD). (F) IMCL deposition in the GP muscle complex. Data are mean ± SEM. Statistics: (A–C, F) Two-way ANOVA (repeated measures used for A–C) with Bonferroni post-tests; (D–E) student’s t-test, *p<0.05. ND, white bars; HFD, black bars unless noted.

Following either an overnight or a 6 hour fast an intraperitoneal glucose tolerance test (IPGTT) was performed. To maximally raise blood glucose, stress the system’s responsiveness to a glucose challenge and to assess insulin sensitivity in muscle, a higher dose of glucose was employed following the overnight fast. Glucose tolerance was impaired (Figure 3A, B) with HFD after 3 weeks as determined by IPGTT. This occurred despite similar baseline glycaemia between diet groups. During an intraperitoneal insulin tolerance test (IPITT; 6 hr fast), whole-body insulin sensitivity was not different with HFD (Figure 3C). The presence of glucose intolerance in HFD mice (Figure 3A, B) could involve a reduced ability of adipose and muscle to uptake glucose in response to insulin secreted by the pancreas. However, as insulin-stimulated glucose uptake in isolated EDL (Figure 3D) and soleus (Figure 3E) muscles was maintained in HFD fed mice our findings suggest that the glucose intolerance was not due to the presence of skeletal muscle insulin resistance. Thus, glucose intolerance in HFD mice is likely due to a lower responsiveness of the liver to insulin which should translate to a slower lowering of blood glucose during an ITT. The normal ITT result in HFD mice may be a result of initial hyperinsulinemia at baseline although this was not assessed. The lack of muscle insulin resistance coincided with elevated total palmitate oxidation and unaltered IMCL content observed in the mixed fiber-type GP muscle complex (Figure 3F). Disease progression was appropriately worsened with time, since plasma insulin levels were not different from control mice following 3 weeks of HFD, but were ∼4 fold higher following 8 weeks of HFD (Figure S2).

### Increased Inflammation in Adipose Tissue, but Little to no Change in Liver, Muscle or Blood Following 3 Weeks of HFD

**Figure 4:**
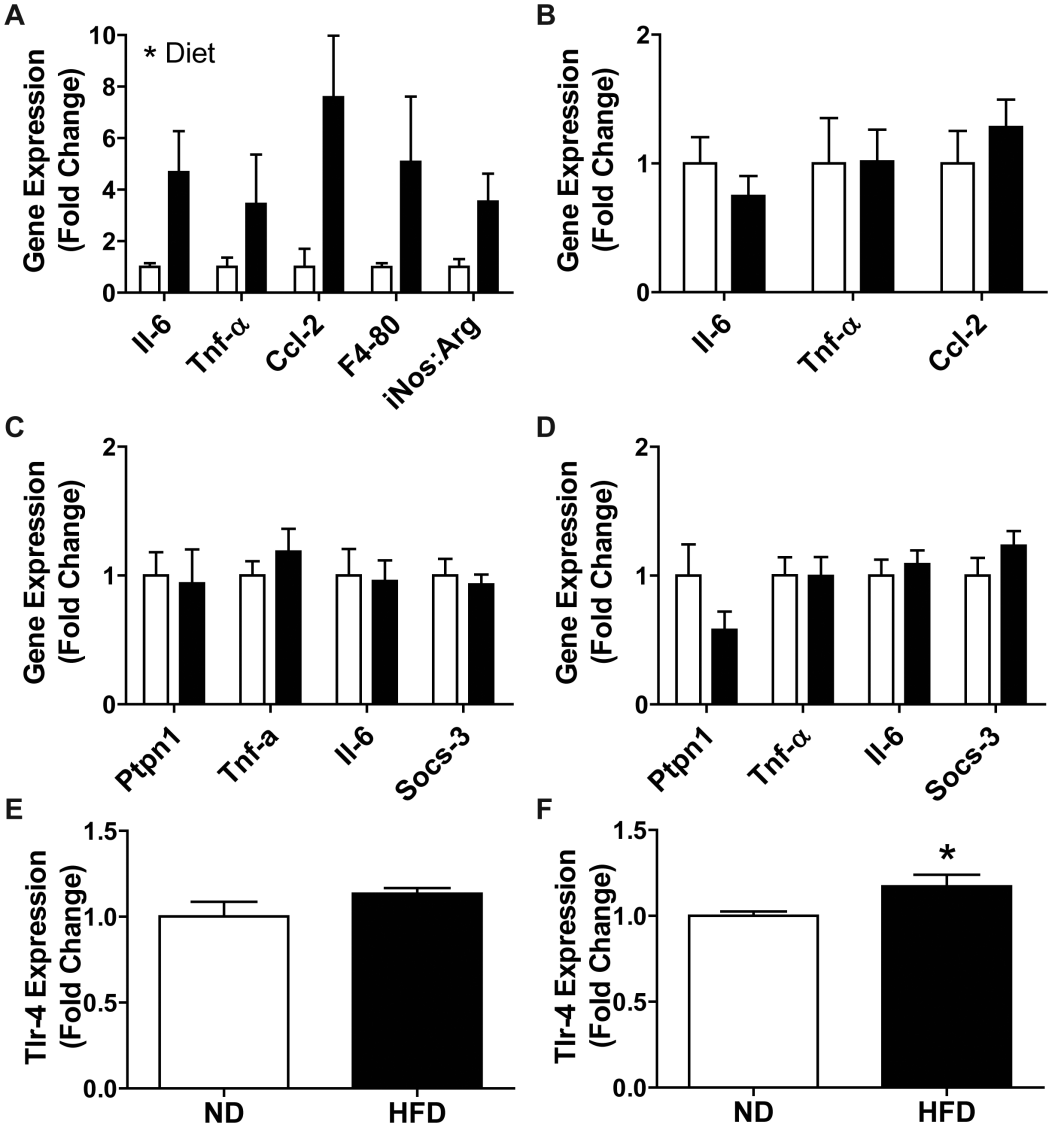
Indices of adipose, liver, blood and muscle inflammation following 3 weeks of HFD. Expression of pro-inflammatory indices in (A) adipose tissue (N = 3–6), (B) liver (N = 5–6), (C) TA muscle (N = 6) and (D) soleus muscle (N = 5 ND and 6 HFD). *Tlr-4* gene expression in (E) TA (N = 6) and (F) soleus muscle (N = 5 ND and 6 HFD). Data are means ± SEM. Statistics: (A–D) All ND data was normalized to 1, Two-way ANOVA with Bonferroni post-tests; (E–F) student’s t-test, * p<0.05. Values were normalized to β-actin (muscle and adipose tissue) or TATA binding protein (TBP; liver tissue). Normal diet (ND, white bars), high-fat diet (HFD; black bars).

Lipid overload and inflammation in adipose tissue and liver is linked to the onset of insulin resistance in these organs [16], [17], [27], [28]. The significant adipose tissue expansion observed after 3 weeks of HFD (Table 1) was accompanied by an increase in pro-inflammatory markers in this tissue (Figure 4A), including the iNOS:arginase ratio, which is indicative of pro-inflammatory M1 macrophages [29]–[31]. While there was a significant increase in hepatic lipid deposition (ND 0.495±0.08%area; HFD 1.211±0.21%area, N = 3–5), pro-inflammatory indices in the liver were unaltered (Figure 4B). Inflammatory proteins produced by adipose tissue can be released into the circulation where they can act on alternative insulin sensitive tissues like skeletal muscle. However, there were no alterations to a wide range of pro- and anti-inflammatory factors in the plasma of HFD mice including IL-6, TNF-α, and CCL-2 (Figure S3A). There was also a lack of overt inflammation in skeletal muscle after 3 weeks of HFD as evidenced by unaltered macrophage content, identified by the F4–80 glycoprotein in GP muscle (ND 11.75±1.88 AU; HFD 19.00±6.24 AU, N = 3–4). Moreover, mRNA expression of inflammatory genes including *Socs3*, *Il-6*, *Tnf-α* and *Ptp-n1* in skeletal muscle were found to be unaltered in both tibialis anterior (TA) and soleus muscles (Figure 4C, D). Inflammatory signaling within skeletal muscle could also be triggered by circulating fatty acids through toll-like receptor-4 (*Tlr-4*) which was elevated in soleus, but not TA muscles (Figure 4E, F), following 3 weeks of HFD.

### 8 Weeks of HFD Results in Minor Alterations to Inflammatory Signaling in Muscle

**Figure 5:**
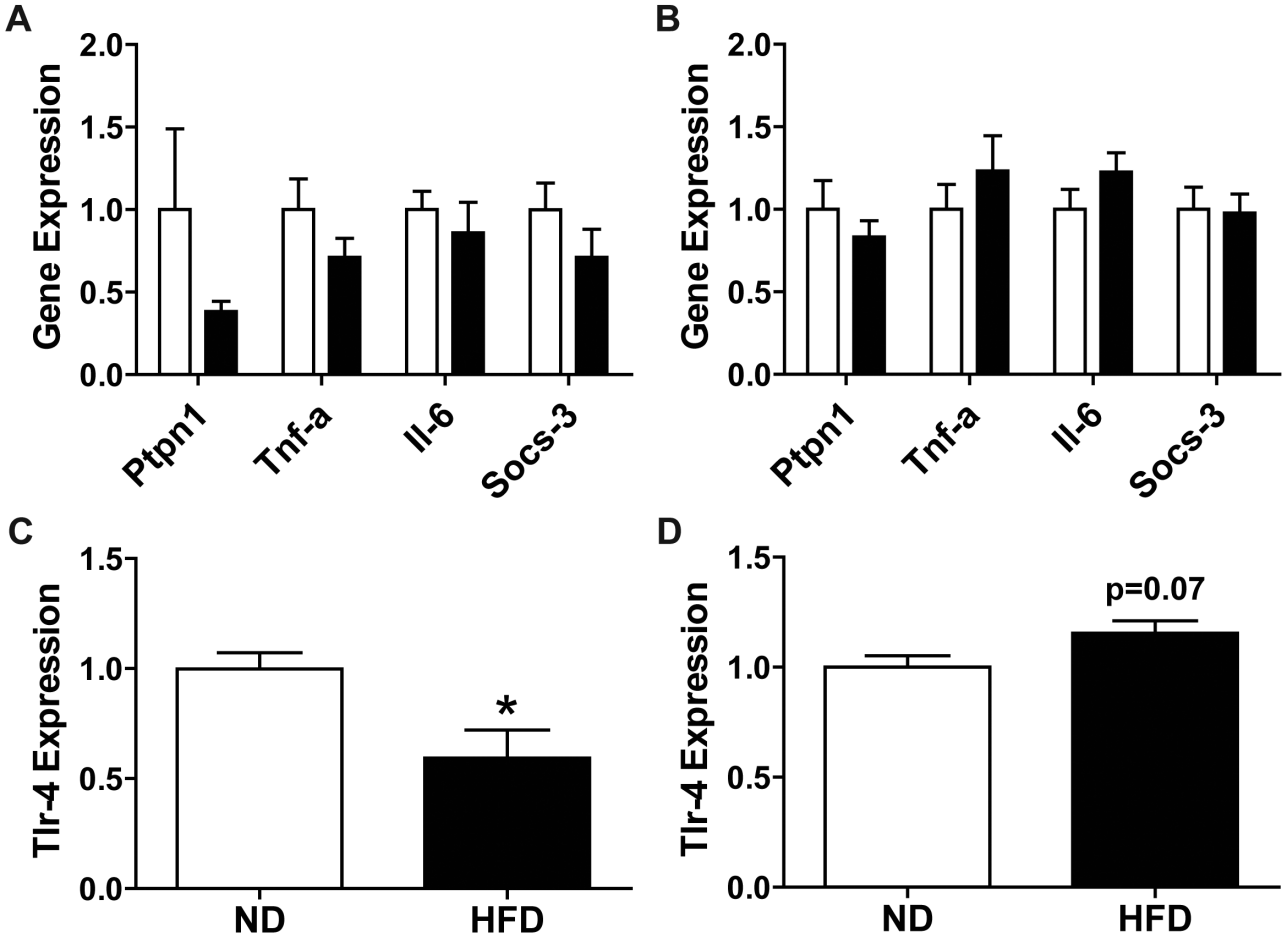
Pro-inflammatory transcripts in muscle following 8 weeks of HFD. Relative expression of pro-inflammatory transcripts in (A) TA and (B) soleus muscle after 8 weeks of HFD. Relative expression of *Tlr-4* in (C) TA and (D) soleus muscle after 8 weeks of HFD. Values were corrected for β-actin expression and ND values were normalized to a value of 1. Statistics: (A and B) Two-way ANOVA with Bonferroni post-tests; (C and D) student’s t-test, *p<0.05. Normal diet (ND, white bars), high-fat diet (HFD; black bars).

Since skeletal muscle insulin resistance has been reported after 8 weeks of our HFD [18] and pro-inflammatory factors in plasma have been implicated in the development of skeletal muscle insulin resistance [15], [17], inflammatory markers in the plasma and inflammatory signaling in skeletal muscle were investigated at this time-point. There was no difference in plasma inflammatory proteins including Il-6, Tnf-α and Ccl-2 following 8 weeks of HFD (Figure S3B). The majority of inflammatory signaling in muscle was found to be unaltered with HFD in TA or soleus muscles (Figure 5 A, B). However, *Tlr-4* expression was down-regulated in TA muscle and there was a trend (p = 0.07) towards an increase in soleus muscle (Figure 5 C, D), which is similar to 3 weeks of HFD.

## Discussion

Our results indicate that the early, whole body response to HFD, particularly that of skeletal muscle, is a protective adaptation which provides a buffering period of time before the onset of insulin resistance in muscle after 8 weeks of HFD as previously demonstrated in this model [18]. Specifically, we found that HFD resulted in an early shift towards enhanced whole body and total muscle lipid oxidation and an oxidative fiber-type shift, which we believe attenuated IMCL build-up and prevented muscle insulin resistance following 3 weeks of HFD. Unfortunately, enhanced total lipid oxidation was unable to prevent glucose intolerance which was present after 3 weeks of HFD and associated with adipose tissue inflammation, increased adipose tissue mass and elevated hepatic lipids. Knowing that muscle is insulin resistant after 8 weeks of our HFD treatment [18], the minimal elevations of inflammatory markers identified in muscle and plasma at, and prior to, this time-point leads us to speculate that inflammation plays a minimal role in the initiation of muscle insulin resistance.

As expected, HFD resulted in a rapid shift towards greater whole body lipid utilization as seen by the reduction in RER within 12–24 hours of HFD commencement. Continued HFD may result in a further reduction of RER as noted in rats fed a HFD [32]. However humans given a HFD experience a rapid reduction in respiratory quotient (RQ) after 24 hours that is not reduced further following 7 days [33]. The reduction in RER seen with HFD was not solely due to a reduction in activity level as light cycle activity level was not altered and dark cycle activity level was only reduced temporarily. Total calories consumed were unaltered with HFD demonstrating that the composition of the diet, not a caloric increase, was responsible for the change in metabolism. The shift towards increased total lipid oxidation that occurs within hours of switching to HFD continues at 3 weeks as evidenced by the increase in total palmitate oxidation in the mixed fiber-type EDL muscle, but not the highly oxidative soleus muscle. We hypothesize that the soleus does not exhibit an increase in total lipid oxidation due to the high rate of basal (e.g. ND) total lipid oxidation compared to EDL muscle. The rapid increase in IMCLs observed with lipid infusion or HFD [32] appears to have provided the stimulus for increased muscle fatty acid oxidation in EDL muscle by 3 weeks of HFD which, in turn, helped maintain IMCLs at levels similar to those of ND mice. Conversely we previously demonstrated prolonged HFD (8 weeks) to result in impaired lipid oxidation, excess IMCLs and insulin resistance in muscle [18].

In line with unaltered IMCL levels in the GP muscle, EDL muscles were insulin-sensitive and exhibited a lower esterification-to-oxidation ratio following 3 weeks of HFD. A high esterification-to-oxidation ratio is observed in obese, insulin resistant states and is thought to contribute to both the build-up of lipids and insulin resistance in muscle [33], [34]. An increase in the ratio of incomplete to complete fatty acid oxidation has also been proposed to contribute to muscle insulin resistance [35]. Accordingly, this ratio was decreased for both muscle types in the present study. In the present study whole muscle, as opposed to muscle homogenates, was used to assess palmitate oxidation resulting in the majority of oxidative intermediates, representative of incomplete oxidation, remaining within the muscle and few being released into the surrounding media. Thus, oxidative intermediates (termed ASMs when using homogenates) trapped within muscle were assessed here whereas with homogenates ASMs in the media are determined. Given unaltered palmitate uptake for both muscle types, yet a lower esterification to oxidation ratio for EDL muscle alone, we conclude HFD caused EDL muscle to exhibit a greater rate of fatty acid uptake into mitochondria. There have been differing reports in the literature concerning muscle lipid oxidation with HFD [18], [20], [21], [36], [37]. An initial increase in muscle lipid oxidation has been reported in a few studies [20], [21] which is consistent with our findings after 3 weeks of HFD. Importantly our enhanced lipid oxidation at 3 weeks demonstrates an early change to muscle in response to HFD that may have mitigated the build-up of IMCLs within muscle, which we believe is the mechanism underlying the maintenance of muscle insulin sensitivity after 3 weeks of HFD.

Our reported increase in muscle lipid oxidation was likely due to a rapid decrease in pyruvate dehydrogenase that is known to occur with a low-carbohydrate and high-fat diet [38]. A prolonged elevation in lipid oxidation could result in a shift in muscle fiber-types or mitochondrial density. No increase in density of the mitochondrial marker, SDH, was found in oxidative fibers which we previously noted after 8 weeks of HFD [18]. However, an increase in oxidative capacity was evident in muscle after 3 weeks of HFD by an oxidative fiber-type shift within the oxidative area of the GP muscle as was also noted following our previous 8 week HFD study [18]. Although our assessment of fiber-type content is sensitive to early changes in oxidative capacity, our assessment of whole muscle OXPHOS content provides a more global picture of the change in oxidative capacity in the predominantly fast glycolytic GP muscle [26]. Our findings suggest an early adaptive response to HFD within muscle by upregulating lipid oxidation and transitioning to a more oxidative fiber-type. We have now shown that prolonged exposure to HFD (8 weeks) results in further enhancement of the oxidative potential of muscle as demonstrated by an increase in OXPHOS content which complements our previous report of an oxidative fiber type shift after 8 weeks of HFD [18]. However, at the 8 week time-point we previously demonstrated functional lipid oxidation to be impaired with this diet paradigm [18]. Thus, there is an early increase in metabolic machinery that coincides with an increased functional ability to oxidize lipids. While there is a further increase in metabolic machinery with prolonged HFD the functionality is reduced. These studies indicate that measuring the expression of proteins involved in oxidative metabolism alone does not necessarily translate into functional lipid oxidation rates in skeletal muscle, thus both measures should be assessed.

Rapid expansion of adipose tissue upon HFD consumption could be seen as a positive adaptation, since it accommodates a larger supply of circulating FFAs, thereby reducing hyperlipidemia and ectopic lipid deposition in other tissues. HFD for 3 weeks resulted in a significant increase in body mass and epididymal fat pad mass with no change in lower limb muscle mass demonstrating significant adipose tissue expansion. Such a large expansion of adipose tissue could involve the conversion of preadipocytes to adipocytes, known as adipogenesis, or enlargement of existing adipocytes which would constitute hypertrophy. Should adipogenesis have occurred, our observed elevation in adipose tissue *Il-6* expression could be derived from insulin stimulated preadipocytes [39]. In contrast, *Tnf-α* expression was elevated in adipose tissue after 3 weeks of HFD and Tnf-α has been shown to cause transcriptional changes in adipose tissue leading to the prevention of preadipocyte differentiation [40]. Hypertrophy of adipose tissue is also associated with inflammatory cell infiltration and activation of M1 macrophages which are needed for ECM remodeling during adipose tissue expansion [30], [41]. We found elevated expression of the F4/80 glycoprotein and the iNOS to arginase ratio demonstrating a greater macrophage content that is of the M1 pro-inflammatory phenotype which secretes Tnf-α, Il-6 and Ccl-2, all of which were found to be upregulated at the mRNA level after 3 weeks of HFD [24]. Ccl-2 serves to recruit new macrophages, while Il-6 and Tnf-α encourage the inflammatory cycle [24]. This data serves to support previous reports associating HFD induced adipose tissue mass expansion and adipose tissue inflammation with glucose intolerance [24]. Interestingly a recent report demonstrated adipose tissue inflammation was not necessary for development of glucose intolerance following 3 days of HFD although it did contribute following a prolonged (16 week) HFD [42]. In the present study, glucose intolerance was present following rapid adipose tissue expansion and adipose tissue inflammation despite enhancement of lipid oxidation and maintenance of muscle insulin sensitivity.

While adipose tissue is capable of adaptation to accommodate excess circulating lipids, the liver has minimal capabilities for lipid accommodation. The liver receives a large amount of lipids due to direct dietary supply from the portal vein and other circulating lipids that are not oxidized by muscles and other tissue. In this context, the liver may be more susceptible than skeletal muscles and adipose tissue to lipotoxicity under conditions of lipid oversupply such as when feeding a HFD. It is known that within 3 days of HFD, and possibly sooner, hepatic lipids increase [42] and we observed this to be true following 3 weeks of HFD. Increased hepatic lipid content causes insulin resistance resulting in elevated gluconeogenesis and reduced glycogen synthesis in the post-prandial state [16], [17]. The liver significantly contributes to glucose uptake during an IPGTT, normally accounting for ∼30–40% [43]. Given our observed increase in hepatic lipids with HFD, early insulin resistance likely occurred in liver and this would contribute to the impaired IPGTT result after 3 weeks of HFD despite insulin sensitivity in muscle. While prolonged HFD consumption has been shown to induce hepatic inflammation, which also contributes to insulin resistance, no overt evidence for an increase in inflammation was noted in liver suggesting that this organ was at an early stage of insulin resistance [23], [44]. Large elevations of inflammatory factors were also absent from plasma after 3 or 8 weeks of HFD. This is in line with a recent study in which neither Il-6 nor Tnf-α were elevated in mice following 12 weeks of HFD [45]. We conclude that inflammation in liver and plasma are not directly associated with early glucose intolerance.

Inflammation in muscle has recently been suggested to contribute to muscle insulin resistance [15]. The only indication of inflammation in muscle was a temporary increase in *Tlr-4* expression after 3 weeks of HFD. Interestingly in addition to cytokines, saturated fatty acids and LPS have been argued to induce an increase in *Tlr-4* expression [46], [47]. While increased muscle *Tlr-4* expression has been shown to contribute to insulin resistance [48], no insulin resistance was present after 3 weeks of HFD when *Tlr-4* was upregulated. Furthermore, our longer 8 week HFD that led to muscle insulin resistance [18] was only associated with a trend towards upregulation of *Tlr-4* in soleus and a significant downregulation of *Tlr-4* in TA muscle. Thus, while the role of Tlr-4 during early development of muscle insulin resistance warrants further investigation it is clear that inflammation in muscle plays a minimal role in the development of early muscle insulin resistance.

In summary we have demonstrated tissue specific changes to gene expression, metabolism, lipid deposition, and inflammation in C57BL/6J mice fed a HFD which develop characteristics of Metabolic Syndrome similar to humans and will eventually develop T2DM including elevated fasting blood glucose [49], [50]. Similar to our results a recent study demonstrated that the pathogenesis of HFD induced glucose intolerance and insulin resistance in mice is characterized by early metabolic and inflammatory changes to adipose and liver tissue [51]. Glucose intolerance was noted with only 1 week of HFD coinciding with our early observation of glucose intolerance [51]. Furthermore, a reduction in transcripts for lipid metabolism was identified in muscle after 6 weeks of HFD which is similar to our previously reported reduction in functional lipid oxidation after 8 weeks of HFD [18]. The present study found that 3 weeks of HFD resulted in early positive adaptations in muscle including enhanced lipid oxidation, an oxidative fiber type shift and maintenance of IMCL levels which would all contribute to maintenance of muscle insulin sensitivity. Unfortunately the positive adaptations within muscle were unable to prevent glucose intolerance which was associated with inflammation in the adipose tissue and the amount of lipids stored in both adipose and hepatic tissues. Only by fully understanding the stages of the onset of a disease can one successfully devise therapies and treatments to delay or prevent its progression. Thus, it is important to understand the underlying etiology of HFD-induced glucose intolerance and muscle insulin resistance. We propose that the adaptations to muscle we observed are advantageous to slowing the development of impaired glucose tolerance. Further studies manipulating HFD-induced adaptations to muscle are required to fully understand their importance. However, our hypothesis is consistent with reports in which interventions that increase oxidative capacity in skeletal muscle (e.g. exercise) can delay or reverse muscle insulin resistance [9], [12], [13].

## Methods

### Animals and Blood Sampling

All experimental protocols were approved by the McMaster University Animal Care Committee in accordance with the Canadian Council for Animal Care guidelines. Male C57BL/6J mice were obtained from Jackson Laboratories (Bar Harbor, ME). Animals were housed in a temperature and humidity-controlled facility with a 12/12 h light/dark cycle and had *ad libitum* access to water and food. Following 1 week acclimatization, animals [10 weeks of age; N = 5–6 per group as noted in Table 1 for the first round of experiments (e.g. group 1) lasting 3 weeks (21 days) and 8 weeks (56 days) including 16 hour fasted IPGTT, body and adipose tissue masses, 8 week muscle mass, all histology/cryo-section analysis except liver tissue, muscle qualitative PCR, and plasma collection which was performed in the fed state between 14∶00 and 16∶00 hrs; N = 8 ND and 10 HFD per group for second round of experiments (e.g. group 2) lasting 3 weeks (21 days) including 6 hour fasted IPGTT and IPITT, CLAMS, 3 week muscle mass, liver histology (Oil-Red-O), adipose and liver tissue quantitative PCR, and *ex vivo* muscle glucose and palmitate experiments] were randomly assigned to either a high fat diet [HFD; TestDiet, cat #58126: energy (kcal/g) from protein (18.3%), fat (60.9%), carbohydrate (20.1%)] or normal diet [LabDiet 5015 Mouse Diet: energy (kcal/g) from protein (20%), fat (25%), carbohydrate (55%)]. Fed state body mass was assessed on a weekly basis between 14∶00 and 16∶00 hrs. An IPGTT was performed on mice fasted (16 or 6 hr) after 18 days of diet intervention. Glucose was injected IP (2 g/kg of body weight for 16 hour fasted mice or 1****g/kg body weight for 6 hour fasted mice) and blood glucose was assessed by tail bleed at various time-points.

### Experimental Procedures

Group 1 mice were weighed then euthanized by cervical dislocation following 3 or 8 weeks of diet and tissues were harvested and either snap frozen or mounted with tissue freezing medium and frozen in isopentane cooled by liquid nitrogen then frozen in liquid nitrogen. Group 2 mice underwent CLAMS analysis at the beginning of the diet intervention (N = 8). Mice were placed in the CLAMS and given 24 hours to acclimatize before measurements were taken every 20 minutes. Food intake (see data analysis for calculation) was recorded every 20 min as the difference between weight of ground food removed from the feeder and weight of ground food in the catch tray under the feeder. Mice were weighed and anaesthetized (ketamine/xylazine) prior to dissection of the soleus and EDL muscles for *ex vivo* glucose and palmitate experiments. Euthanization was performed by cervical dislocation and adipose and liver tissues were removed and snap frozen in liquid nitrogen.

### Insulin-stimulated 2 Deoxy-D-glucose (2DG) Uptake in Isolated Muscles

Following 3 weeks of diet intervention, glycolytic EDL and oxidative soleus muscles were removed from anaesthetized (ketamine/xylazine) mice as described in experimental procedures. 2DG uptake (N = 4 ND, 6 HFD per muscle group) was assessed as previously described [45]. Briefly, EDL and soleus muscles were dissected tendon to tendon and quickly placed in glass vials containing 2 ml of pre-gassed (95% O_2_∶5%CO_2_) Krebs-Hanseleit bicarbonate (KHC) buffer (0.1% BSA and 2 mM pyruvate) and pre-incubated for 20 min. Buffer was then replaced with a similar buffer supplemented with −/+ insulin (400 µU/ml) (Actrapid®, Novo Nordisk A/S, Denmark), and muscles were incubated for a further 30 min, before final incubation step whereby 2DG uptake was measured by replacing existing incubation buffer with a similar buffer containing [^3^H]-2DG (0.5 µCi/ml) (Amersham, GE Healthcare, UK), 2-DG (1 mM) and the extracellular space marker [^14^C]-mannitol (0.20 µCi/ml) (Amersham, GE Healthcare, U.K.) and incubating for 20 min. Muscle-specific insulin sensitivity, as assessed by responsiveness to insulin in promoting 2DG uptake, was determined by homogenizing muscles in 300 µl of ice-cold cell lysis buffer (Tris.HCl (20 mM), NaCl (50 mM), dithiothreitol (2 mM), NaF (50 mM), Triton X-100 (1%), sucrose (250 mM), sodium pyrophosphate (5 mM), leupeptin (4 µg/ml), benzmidine (6 mM), phenylmethylslfonylfluoride (500 µM), soybean trysin inhibitor (50 µg/ml), pH 7.4)) and counting radioactivity in 100 µl of muscle lysates by liquid scintillation counting as described previously [45].

### *Ex vivo* Palmitate Experiments and Extraction of Muscle Lipids

Palmitate oxidation (N = 4 ND, 4 HFD per muscle group, average of duplicates/mouse analyzed) was assessed as previously described [52]. Briefly, soleus and EDL muscles were dissected tendon to tendon and pre-incubated in 2 mL of pre-warmed (30°C) and gassed (95%O_2_∶5%CO_2_) basal buffer (modified Krebs Henseleit buffer with 4% fatty acid-free BSA, 2 mM pyruvate and 0.5 mM palmitate) for 20 min. Incubation media was then replace with a similar buffer containing [^14^C]palmitic acid (0.5 µCi/mL) for 60 min. Upon removal and freezing of muscles in liquid nitrogen for determination of esterification and oxidative intermediates, complete oxidation of palmitate was assessed by the collection of ^14^CO_2_ over 90 minutes in 400 µl benzethonium hydroxide following the addition of 1 mL of acetic acid (1 M).

Muscle lipids were extracted from frozen muscle and quantified as previously described [53]. Briefly, muscles were weighed, homogenized (chloroform:methanol, 2∶1), following a series of centrifugations and the addition of ddH_2_O part of the aqueous phase (1 mL) was removed for quantification of oxidation intermediates and part of the lipophilic phase (650 ul) was removed for lipid quantification. Chloroform was evaporated by N_2_, redissolved in 50 µl (chloroform:methanol, 2∶1), and spotted onto oven-dried silica gel plates (Fisher Scientific Canada, Missisauga, ON). Lipids were resolved by placing the silica plates in a solvent (60∶40:3 heptane-isopropyl ether-acetic acid) containing sealed tank, then plates were air dried, sprayed with dichlorofluorescein dye (0.02% wt/vol in ethanol), visualized with long-wave UV light and bands were scraped into vials for determination of radioactivity by liquid scintillation counting. A cocktail of standards (triacylglycerols and diacylglycerols) was run in a separate lane on the silica plates to ensure correct identification of lipids.

### Histochemical Analysis

Serial sections of GP muscle (8 µm) were cut on a cryostat with one section used for a metachromatic stain to determine fiber type and the other section used for a IIA myosin immunofluorescent (IF) and Oil-Red-O co-stain to confirm fiber type and assess IMCL deposition. Fiber type was assessed in the oxidative region of the GP muscle (N = 4 ND and 3 HFD). IMCL was assessed by fiber type (N = 3–4 ND and 4–5 HFD, minimum 45 fibers per muscle). IIA myosin (2F7, DSHB) and Oil-Red-O was assayed by fixation in 2% PFA followed by regular IF procedure including a MOM blocking kit (Vector Labs), overnight primary antibody incubation (neat, 4°C), Alexa 488 goat anti-mouse secondary (1∶1000, 1 hr at room temperature, Invitrogen), Oil-Red-O incubation (30 min) with rinses and mounted with fluoromount (Sigma). SDH activity was assayed as previously described (18) on a GP muscle x-section in serial to a section on which a IIA myosin IF was performed as described above without the Oil-Red-O step (N = 4 ND; minimum 15 IIA fibers per muscle). Determination of Oil-Ref-O and SDH content in muscle was performed using Nikon Elements (Nikon). Briefly, fibers of interest were circled and the mean density was recorded. For both stains a darker stained fiber implies greater content of SDH activity or IMCL content and both translate to a greater mean density. Liver was stained with Oil-Red-O and a hematoxilyn counter stain and lipid content was determined by the percentage area that contained lipid (e.g. object area fraction) in the whole liver section. All images were acquired with a Nikon Eclipse 90i microscope.

### Western Blotting Analysis

Muscle lysates (20 µg) were run out on a 4–15% gradient gel (BioRad) at 60V and transferred overnight (4°C, 20V). Primary antibody (OXPHOS rodent WB antibody cocktail; Abcam; 1∶1000 dilution) was incubated overnight at 4°C followed by secondary goat anti-mouse HRP (Abcam) and detected with SuperSignal chemiluminescent detection reagent (Thermo Fisher).

### mRNA Analysis

Tissue mRNA was isolated using Trizol reagent (Invitrogen) and converted to cDNA with SSIIRT (Invitrogen). Realtime RT-PCR was performed with cDNA from adipose and liver tissue using on the Rotorgene 3000 (Corbett Research) with assay-on-demand gene expression kits (Applied Biosystems) following the manufacturer’s instructions. Expression levels were calculated using the comparative critical threshold (C_t_) method. Semi-quantitative RT-PCR was performed with cDNA from muscle as previously described [54] using the following primer sets: *Socs-3*, TaqMan Mm01249143_g1**;** *Il-6*, 5′-GACAAAGCCAGAGTCCTTCAGAG-3′ 5′-CTAGGTTTGCCGAGTAGATCTC-3′; *Tnf-,* 5′-TCGTAGCAAACCACCAAGTG-3′ 5′GGAGTAGACAAGGTACAACC-3′; *Tlr-4*, 5′-CACTGTTCTTCTCCTGCCTGAC-3′ 5′-CCTGGGGAAAAACTCTGGATAG-3; *Ptpn1*, 5′-CCCGGCCACCCAAACGCACACT-3′ 5′- GACGCCGCAGACCGCATCCTAAGC-3′.

### Plasma Analysis

Plasma was assessed for 40 inflammatory related proteins using a Proteome Profiler Antibody Array (R&D, Mouse Cytokine Antibody Array, Panel A) as per manufacturer’s instructions.

### Data Analysis

Total calories consumed in CLAMS was calculated as follows: food intake (g/day)×9 kcal/g (fat)×ratio of kcal/g fat in diet (0.6 HFD and 0.25 ND)+food intake (g/day)×4 kcal/g (protein and carbohydrate)×ratio of kcal/g in diet (0.4 HFD and 0.75 ND). All statistical analyses were performed with GraphPad Prism 5 software. Differences between groups were determined using the appropriate student t-test or ANOVA followed by the appropriate post-hoc test. P values less than 0.05 were considered significant. All data presented are mean ± standard error of the mean (SEM).

## Supporting Information

Figure S1 **Body mass and exploratory activity upon diet transition.** (A) Body mass prior to placement of mice in the CLAMS (Pre-ND), 4 days later before the start of HFD (Post-ND) and after 4 days of HFD (Post-HFD). (B) Light-cycle and (C) dark cycle exploratory activity. Data are mean ± SEM. B–C: Normal diet (white bars), high-fat diet (black bars). Repeated measures one-way ANOVA with Tukey’s multiple comparison test, p<0.05. Average CLAMS measurements are means of measurements taken every 20 minutes with 1 mouse/CLAMS cage and 8 cages total. Light cycle = 07∶00–19∶00, dark cycle = 19∶00–07∶00.
(TIF)

Figure S2 **Plasma insulin during diet protocol.** Fed state insulin values prior to and following 3 and 8 weeks of diet intervention. Values are mean ± SEM, two-way ANOVA with Bonferroni post-tests, *P<0.05. Normal diet (white bars), high-fat diet (black bars).
(TIF)

Figure S3 **Plasma inflammation indicators.** An antibody panel assessing 40 inflammatory proteins was performed on fed state plasma collected during the light cycle after (A) 3 and (B) 8 weeks of diet intervention. ND values (N = 3) were normalized to 1 and HFD values (N = 3, grey bars) were plotted (mean ± SEM). Inflammatory factors with SEM bars that did not visibly interact with the value of 1 (3-week: BCL, G-CSF, IL-1a, IL-5, IL-7, IL-10, KC; 8 week: BCL, IL-1a, IL-2, M-CSF, TIMP-1) were assessed using a t-test. None of the t-test results were significant (data not shown). In addition IL-6, TNF-α, CCL-2 and IL-10 were assessed using a two-way ANOVA with Bonferroni post-tests, p<0.05. There were no significant results from any of the two-way ANOVAs performed (data not shown).
(TIF)

Table S1 **Muscle mass**. Lower limb muscle mass after 3 and 8 weeks of diet intervention. Tibialis anterior (TA), gastrocnemius/plantaris complex (GP), normal diet (ND), high-fat diet (HFD). Values are mean ± SEM in mg, t-test comparing diet groups for each muscle and time-point.
(TIF)
