# Supplementary figures and images for: Enhanced Lipid Oxidation and Maintenance of Muscle Insulin Sensitivity Despite Glucose Intolerance in a Diet-Induced Obesity Mouse Model
Source: PLoS One. 2013 Aug 12;8(8):e71747. doi: 10.1371/journal.pone.0071747 (PMC3741110; doi:10.1371/journal.pone.0071747)

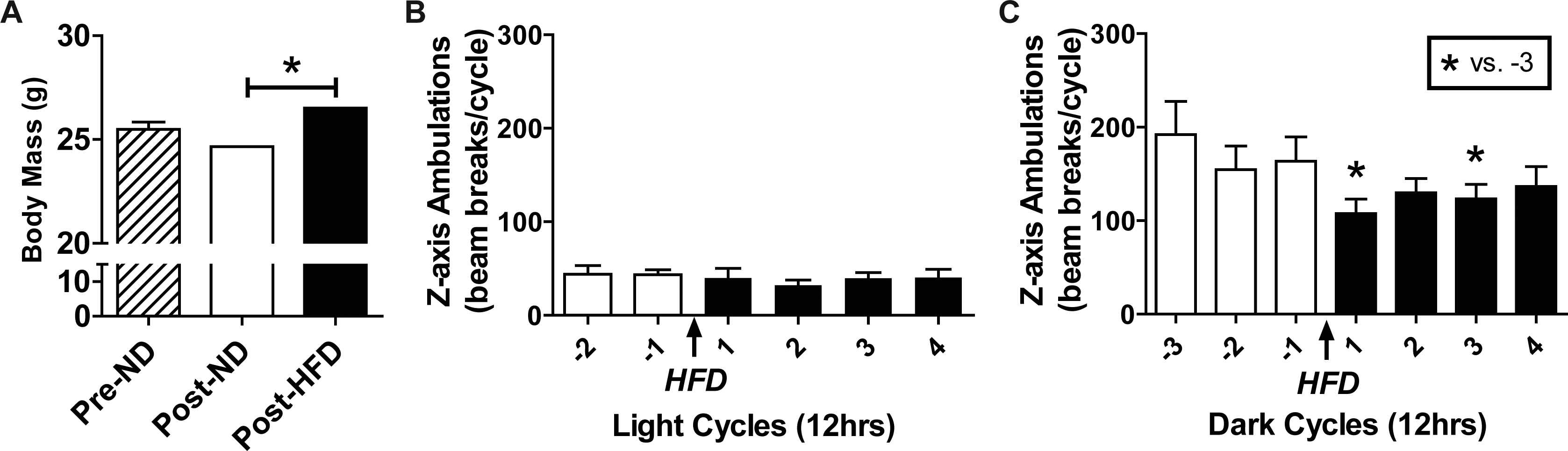

Supplement: Figure S1 — Body mass and exploratory activity upon diet transition. (A) Body mass prior to placement of mice in the CLAMS (Pre-ND), 4 days later before the start of HFD (Post-ND) and after 4 days of HFD (Post-HFD). (B) Light-cycle and (C) dark cycle exploratory activity. Data are mean ± SEM. B–C: Normal diet (white bars), high-fat diet (black bars). Repeated measures one-way ANOVA with Tukey’s multiple comparison test, p<0.05. Average CLAMS measurements are means of measurements taken every 20 minutes with 1 mouse/CLAMS cage and 8 cages total. Light cycle = 07∶00–19∶00, dark cycle = 19∶00–07∶00. (TIF) [file pone.0071747.s001.tif]

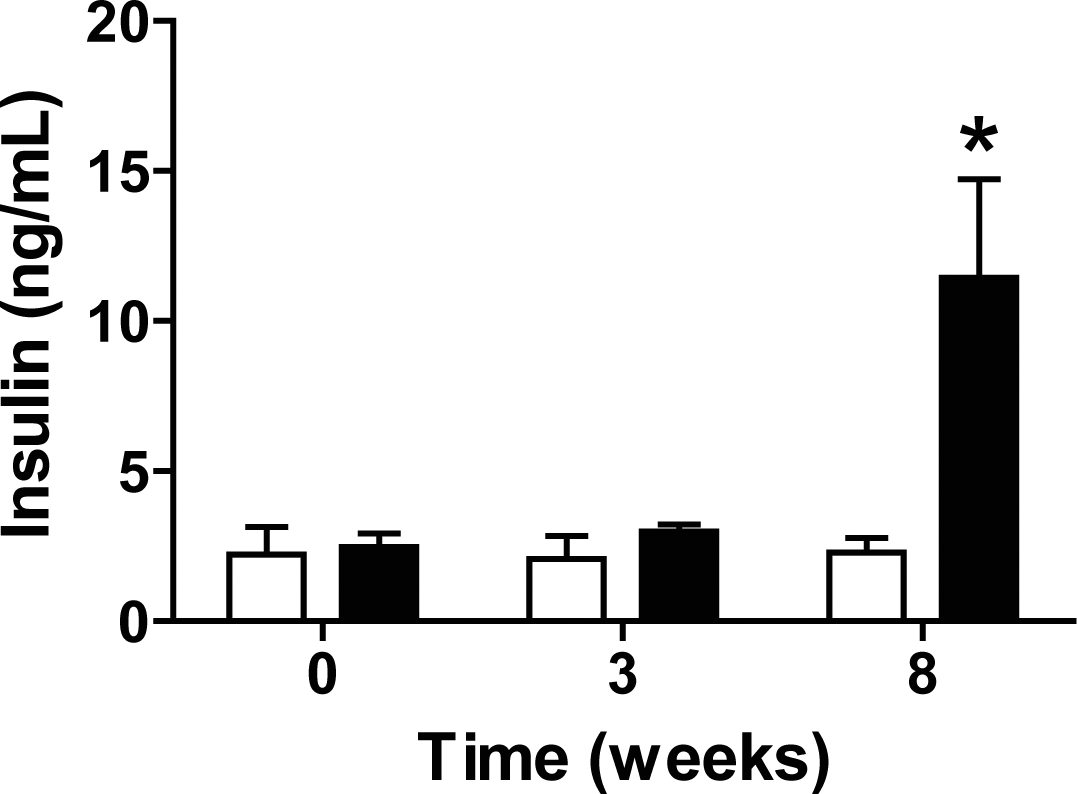

Supplement: Figure S2 — Plasma insulin during diet protocol. Fed state insulin values prior to and following 3 and 8 weeks of diet intervention. Values are mean ± SEM, two-way ANOVA with Bonferroni post-tests, *P<0.05. Normal diet (white bars), high-fat diet (black bars). (TIF) [file pone.0071747.s002.tif]

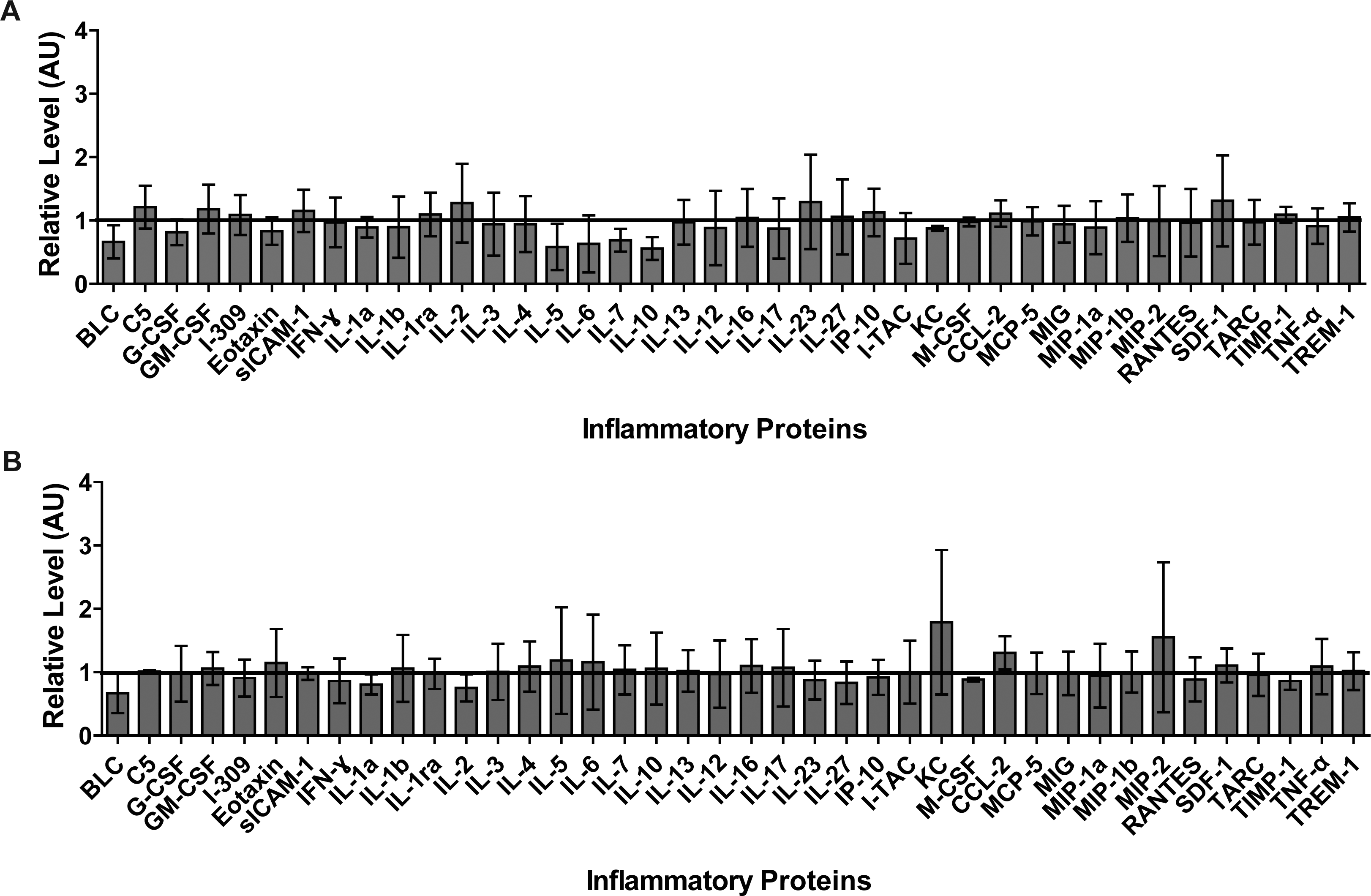

Supplement: Figure S3 — Plasma inflammation indicators. An antibody panel assessing 40 inflammatory proteins was performed on fed state plasma collected during the light cycle after (A) 3 and (B) 8 weeks of diet intervention. ND values (N = 3) were normalized to 1 and HFD values (N = 3, grey bars) were plotted (mean ± SEM). Inflammatory factors with SEM bars that did not visibly interact with the value of 1 (3-week: BCL, G-CSF, IL-1a, IL-5, IL-7, IL-10, KC; 8 week: BCL, IL-1a, IL-2, M-CSF, TIMP-1) were assessed using a t-test. None of the t-test results were significant (data not shown). In addition IL-6, TNF-α, CCL-2 and IL-10 were assessed using a two-way ANOVA with Bonferroni post-tests, p<0.05. There were no significant results from any of the two-way ANOVAs performed (data not shown). (TIF) [file pone.0071747.s003.tif]

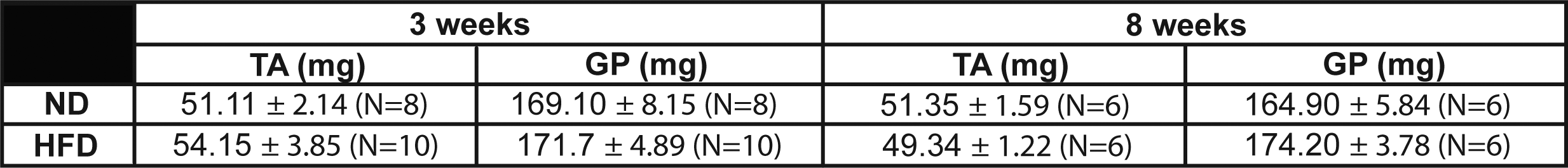

Supplement: Table S1 — Muscle mass. Lower limb muscle mass after 3 and 8 weeks of diet intervention. Tibialis anterior (TA), gastrocnemius/plantaris complex (GP), normal diet (ND), high-fat diet (HFD). Values are mean ± SEM in mg, t-test comparing diet groups for each muscle and time-point. (TIF) [file pone.0071747.s004.tif]
